# The Results of Vascular and Biliary Variations in Turks Liver Donors: Comparison with Others

**DOI:** 10.5402/2011/367083

**Published:** 2011-08-28

**Authors:** Mustafa Ozsoy, Murat Zeytunlu, Murat Kilic, Mehmet Alper, Murat Sozbilen

**Affiliations:** ^1^Department of General Surgery, Manisa Merkezefendi State Hospital, 45000 Manisa, Turkey; ^2^Department of Surgery, Liver Transplantation and Research Center, International Kent Hospital, Izmir, Turkey; ^3^Department of General Surgery, Organ Transplantation and Research Center, Ege University School of Medicine, Izmir, Turkey

## Abstract

*Objective*. To evaluate liver anatomy with a view to access unerring surgery in liver donors. *Summary Background Data*. Liver transplantation, the unique curative treatment option for end-stage hepatic failure, has become routinely practicable, which was inconceivable in the past. But, the vascular and biliary anatomy of the liver has not been completely disclosed yet. *Methods*. From 1994 to 2009, we have done a research on 496 liver donors. The data were accumulated and categorized according to the most widely used classification systems. *Results*. Of 496 liver donors, 393 (79.1%) underwent the right donor hepatectomy, 98 (19.9%) were performed the left lateral segmentectomy, and 5 donors (1%) underwent the left donor hepatectomy surgery. Given the data regarding to 398 liver donors undergone right and left donor hepatectomy, arteries, bile ducts, and portal vein showed classical anatomy in 107 (21.6%) donors. Variations in all three systems were found in 16 donors (3.2%). In the remaining 275 donors (75.2%), anatomical variations were found at either of arterial, biliary, or portal system. *Conclusions*. Our study could come up to actual estimate in liver anatomy as any of donors have not been removed in our institute due to high hilar dissection technique.

## 1. Introduction

The liver is the largest gland in the body, which weights between 1200 and 1600 g representing 2% of total body weight [1]. The preliminary information about liver anatomy and its ability to regenerate reaches out to the myth of Prometheus punished by the gods in Greek mythology. Thanks to the researches on rodents that were conducted by Taub [2], the first successful liver resection was done by McClusky III et al. [3] in 1997. The surgeons enhancing their experience with time realized that intrahepatic anatomy as well as macroscopic anatomy of the liver must have been known. Rex and Cantlie are the researchers who pioneered with their studies in that field [4, 5].

Today, with the knowledge gained from oncologic surgical interventions, liver transplantation, the unique curative treatment option for end-stage hepatic failure, has become routinely practicable, which was inconceivable in the past. In brief, liver transplantation is classified in two types: cadaveric liver transplantation, in which entire liver is transplanted and living donor liver transplantation, in which a portion of liver tissue obtained from living donor is transplanted [6]. Living donor transplantation has marked advantages such as directly available organ number, low rates of morbidity and mortality, and graft quality over cadaveric transplantations [6–10]. However, the most important disadvantage is to make the donors who do not have any health problem and are willing to give a new chance to live to the recipient at risk to die in a short period of time be subjected to a major surgery with the risk of mortality [11]. The vascular and biliary anatomy of the liver has not been completely disclosed yet. The anatomic variants of bile ducts (25%–60%) are the most common; they are followed by hepatic artery (30%–50%) and hepatic vein variants. Portal vein variants (15%–30%) are the least frequent. With this study, we aimed to reduce donor morbidity and mortality through contribution of additional information to vascular and biliary anatomy and to provide further knowledge for oncologic hepatobiliary surgeons.

## 2. Materials and Method

This study included liver donors underwent donor hepatectomy surgery in the period from 1994 to 2009 at the Department of Organ Transplantation affiliated with Ege University School of Medicine Department of General Surgery. The potential liver donors were subject to tests according to the steps of elimination of donors defined by Trotter [8]. Among these potential donors, 496 persons who positively passed elimination steps underwent various donor hepatectomy procedures. Donor hepatectomy procedures involve the right hepatectomy, the left hepatectomy, and the left lateral segmentectomy. The demographic data (age, gender, consanguinity, etc.) belonging to the donors were recorded. For donor's volumetric analysis, imaging of vascular system, and to detect probable variants, 3-D multislice abdominal computerized tomography (Ge Sytec Srı; General Electric Medical Systems. Milwaukee. Wisc), color Doppler ultrasonography of portal vein (Siemens Sonoline G60S scanner, Siemens Medical Systems, Erlangen, Germany), and hepatic angiography were used. To image bile ducts, magnetic resonance cholangiopancreatography (1.5 Tesla Magnet power; Magneton Vision, Siemens, Erlangen, Germany) and/or intraoperative cholangiography was used. The findings obtained from the imaging methods and intraoperative particulars were recorded on a database established with Microsoft Excel software.

The data were categorized according to Michels' classification for hepatic artery variants and according to the classification defined by Cheng et al. for portal vein confluence variations. Soyer's modified hepatic vein classification was used for hepatic vein variants; Couinaud classification was used for variations of bile ducts. Statistical analysis was done using statistical package for social sciences software ver.15.0. Chi-square test (Fisher's exact test) was used for statistical analyses between the groups. A *P* value < 0.05 was set to be statistically significant.

## 3. Results

Of 496 liver donors included in the study, 253 (51%) were men and 243 (49%) were women. The ages of liver donors ranged from 18 to 64 years. The mean age of male donors was 29 years; the mean age of female donors was found to be 32 years. Of 496 liver donors, 393 (79.1%) underwent the right donor hepatectomy, 98 (19.9%) were performed the left lateral segmentectomy, and 5 donors (1%) underwent the left donor hepatectomy surgery.

 Given the data regarding to 398 liver donors undergone right and left donor hepatectomy, arteries, bile ducts, and portal vein showed classical anatomy in 107 (21.6%) donors. Variations in all three systems were found in 16 donors (3.2%). In the remaining 275 donors (75.2%), anatomical variations were found at either of arterial, biliary, or portal system.

### 3.1. Variations of Hepatic Artery

The variations of hepatic artery seen in 496 liver donors were classified according to Michels' classification.

**Type 1 (*n*: 320)**

**Figure 1:**
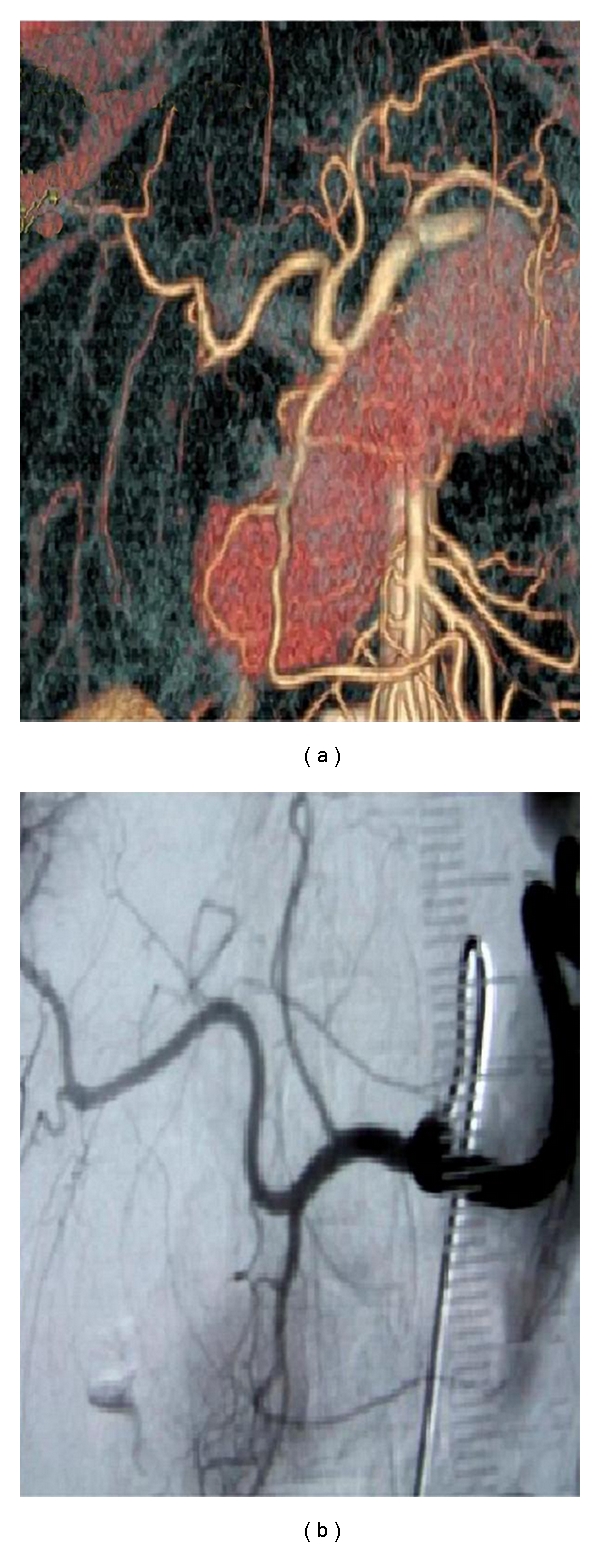
The examples of variation in arterial anatomy. (a) Normal anatomy → Type 1, (b) the replaced left hepatic artery → Type 5.

It is known as normal anatomy (Figure 1(a)). Common hepatic artery arises from the celiac trunk. After forming its gastroduodenal branch, the common hepatic artery is called arteria hepatica propria, which lies over portal vein, usually beneath the choledochus, penetrating at the superior corner of the pancreas and at the left inferior corner of the hepatoduodenal ligament. It divides into two branches, the left and the right arteries, which provide blood supply to the liver.

**Type 2 (*n*: 11)**

The replaced left hepatic artery arises from the left gastric artery (Figure 1(b)). It passes throughout the hepatogastric ligament and enters into the liver through the ligamentum venosum.

**Type 3 (*n*: 43)**

The replaced right hepatic artery arises from the superior mesenteric artery. It enters into hepatoduodenal ligament at inferior and lateral to the choledochus. It lies on lateral to vena porta and penetrates into the liver through the right portal fissure.

**Type 4 (*n*: 7)**

The association of the replaced left hepatic artery and the replaced right hepatic artery.

**Type 5 (*n*: 36)**

Accessory left hepatic artery.

**Type 6 (*n*: 22)**

Accessory right hepatic artery.

**Type 7 (*n*: 4)**

The association of the accessory left hepatic artery and accessory right hepatic artery.

**Type 8 (*n*: 9)**

The association of the replaced right hepatic artery and the accessory left hepatic artery or the association of the accessory right hepatic artery and the replaced left hepatic artery.

**Type 9 (*n*: 9)**

The arteria hepatica propria originated from the superior mesenteric artery.

**Type 10 (*n*: 0)**

The common hepatic artery arisen from the left gastric artery.

**Type 11 (*n*: 39)**

The group named “others” in Michels' classification.

In our study, the proportion of the group that was defined as Type 11 and not classified in Michels' classification was found to be high than the ratio in the original classification. We recognize early branching of the right and left hepatic artery in 33 of 39 patients. That common hepatic artery proceeds as arteria hepatica propria after forming its gastroduodenal artery branch was not observed in 33 (6.6%) donors. The right and left hepatic arteries arise from the common hepatic artery without formation of the arteria hepatica propria. The right and left hepatic arteries directly arose from the aorta in two of the remaining 6 donors, the right hepatic artery arose from the gastroduodenal artery in one donor, the replaced left hepatic artery was originated from the aorta in one donor, the common hepatic artery arose from the SMA in one donor, and the accessory right hepatic artery was directly originated from the aorta in one donor.

### 3.2. Variations of Hepatic Artery

The variations of portal vein were categorized by Cheng classification.

**Type 1 (*n*: 390)**

→ Typical anatomy was seen in 78.6% of the donors. (The main portal vein lies posteriorly within the hepatoduodenal ligament and bifurcates in two branches, the right and the left branches).

**Type 2 (*n*: 63)**

**Figure 2:**
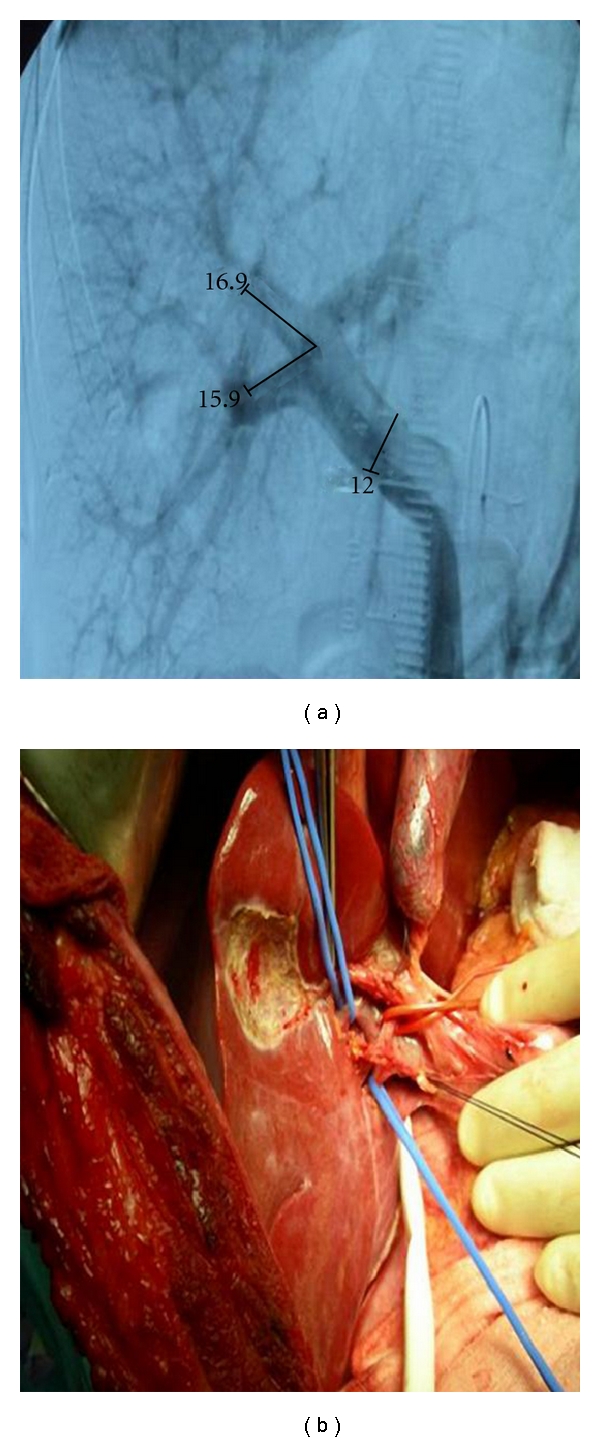
The examples of variation in portal vein anatomy. (a) Trifurcation (the main portal vein divides into three branches), (b) low insertion of the right posterior portal vein.

→ Trifurcation was observed in 12.7% of the donors (Figure 2(a)). (The main portal vein divides into three branches, the right anterior, right posterior, and the left portal veins).

**Type 3 (*n*: 34)**

→ Low insertion of the right posterior portal vein originating from the main portal vein was found in 6.9% of the donors (Figure 2(b)).

**Type 4 (*n*: 9)**

→ The right anterior portal vein arisen from the left portal vein or the left umbilical port was seen in 1.8% of the donors.

### 3.3. Variations of Hepatic Artery

**Figure 3:**
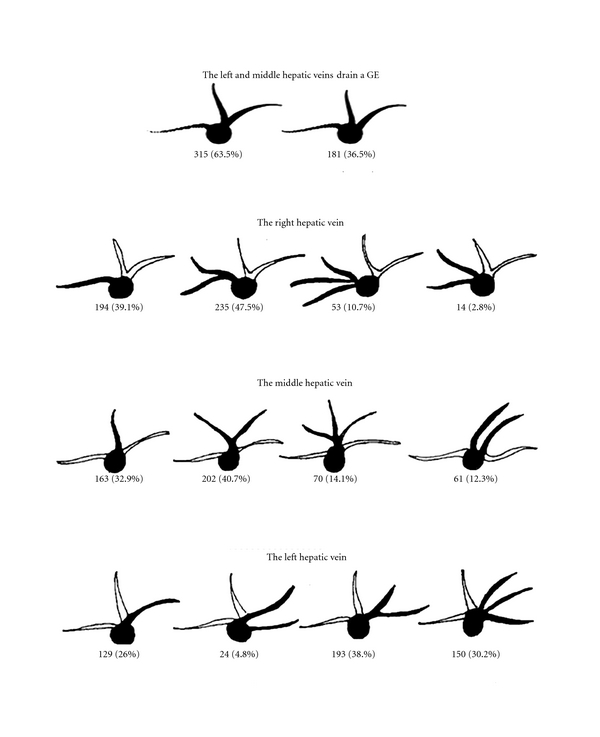
The hepatic vein system's data of 496 liver donors.

The data of 496 liver donors were categorized based on the modified hepatic vein classification defined by Soyer et al. (Figure 3).

### 3.4. Variations of Hepatic Artery

**Figure 4:**
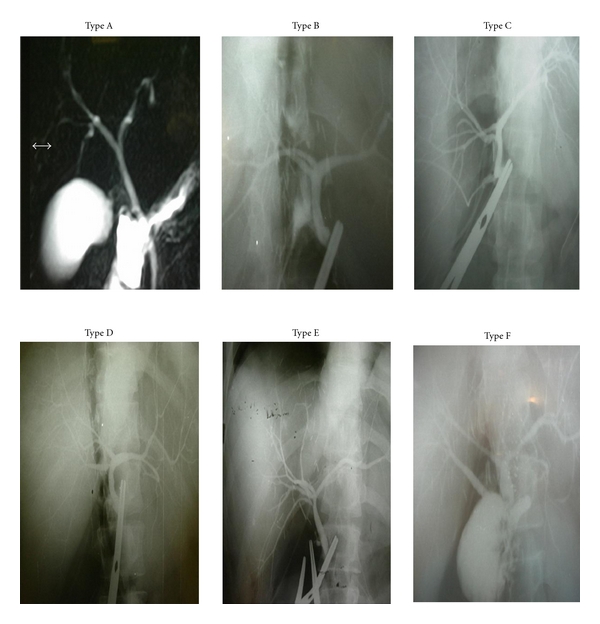
The examples of variations in intra hepatic bile ducts.

The anatomic variations of intra hepatic bile ducts defined by Couinaud in 1957 are still the most commonly used classification system today. Based on this classification system, the data of 398 liver donors underwent right and left liver lobe donor hepatectomy procedure, and the examples of variations were presented below (Table 1, Figure 4).

### 3.5. Variations of Hepatic Artery

**(A) Relationship between Bile Ducts and Variations of Hepatic Artery**

When the variations of hepatic artery and bile ducts seen in 398 liver donors were evaluated, there was no statistical correlation between the artery and bile ducts (*P* : 0.354).

**(B) Relationship between Bile Ducts and Portal Vein Variants**

The relationship between the bile ducts and portal vein variants was evaluated. It was concluded that the probability of coinciding variations of bile duct was high in the subjects with portal vein variants (*P* : 0.019).

**(C) Relationship between Arterial and Portal Vein Variants**

There was no statistical significance between arterial and portal vein variants in the evaluation of 496 cases (*P* : 0.080). Although a clear significance was not found as a consequence of the analyses, it can be commented that the probability of coinciding variations of the other is high when arterial and/or portal vein variants are present.

**(D) Association of Hepatic Vein Variants with Others**

It was not found any statistical correlation between hepatic veins and bile tree (*P* : 0.292), arterial system (*P* : 0.414), and portal system (*P* : 0.131).

## 4. Discussion

### 4.1. Anomalies of Hepatic Artery

With the increasing number of laparoscopic procedures, oncologic surgical interventions, and organ transplant cases, anatomy and variations of hepatic arterial system have become increasingly important. After Michels' autopsy series of 200 cases, several commentaries about extrahepatic arterial anatomy were made in many published articles [13]. It is very important to know about likely hepatic arterial variants to avoid harming particularly the donors in donor surgery and the liver graft on the back table. In the literature, the recognition of arterial variants usually based on angiographic findings only [14]. In our series, preoperative angiography was combined with 3D-CT and intraoperative findings. Arterial system was categorized according to the original Michels' classification. In the present study, intrahepatic arterial anatomy of the liver was not researched. In the light of the published articles in the literature, it has already been reported that intrahepatic arterial system proceeds in parallel to the biliary system and intrahepatic biliary variants are discussed in detail in the following sections of our study. Therefore, the study involved only extrahepatic arterial variants.

Given the results of our series, the proportion of the typical arterial anatomy defined in the anatomy textbooks is consistent with worldwide data [15–17] (Table 2). Our series is of particular importance, since it is the third largest series published by now. Another point that deserves being emphasized is that early branching of hepatic arterial system without formation of the arteria hepatica propria, which is classified in the group of Type 11 in Michels' classification, occurs at considerably high incidence (6.6%).

This study has two limitations. First, we did not search the variations of the cystic artery, because the cystic artery and its variants are not significant for surgical strategies in donor hepatectomy. Further studies are needed about the variations of cystic artery that become important for the surgeons dealing with laparoscopic biliary surgery. The second limitation is that the study involved only a specific group of the subjects, that is, liver donors. From that point of view, although the group seems to be limited and specific, the variations of hepatic artery do not contraindicate to be a donor in our institute because of high hilar dissection technique. Therefore, we think that the results of our study reflect the hepatic arterial variants seen in Turkish population rather than attribute them to a specific group. We hope that the data achieved by this study are useful not only for transplant surgeons, but also for all oncologic hepatobiliary surgeons. 

### 4.2. Anomalies of Hepatic Artery

The variations of the portal vein, which is developed in the early phase of the embryologic period, are rare. In a study with 210 patients, Cheng et al. [18] found typical anatomy (Type 12) in 146 patients (69.52%) and trifurcation anatomy (Type 13) in 40 cases (19.05%). The authors reported the low insertion of the posterior branch of the right portal vein (Type 14 anatomy) in 9 patients (4.29%) and the right portal vein anterior branch arisen from the left portal vein (Type 15) in 15 patients (7.1%). As a result of a sonographic study with 18 500 patients, Fraser-Hill et al. [19] reported that a portal vein variant could be found in one patient in 7 to 10. In a series of 214 patients, Cheng et al. [20] identified Type 12 anatomy in 195 cases (91.1%), Type 13 anatomy in 9 patients (4.2%), Type 14 anatomy in 8 cases (3.7%), and Type 15 anatomy in one patient (0.5%). Atri et al. found variations in 102 (20%) of 507 patients. They found Type 13 anatomy in 55 (11%) of these 102 patients, Type 14 anatomy in 24 cases (5%) and Type 15 variants in 22 (4%) patients [21]. 

Given the results of our series, typical anatomy called Type 12 was found in 390 (78.6%) of 496 potential donors. Type 13 variation, trifurcation anatomy, was observed in 63 (12.7%) donors; Type 14 anatomy was found in 34 (6.9%) subjects; Type 15 variation was seen in 9 (1.8%) donors. The probability of finding variations increases with high number of the subjects. We observe that fact in our series, our rates are consistent with the worldwide literature. It is noteworthy that the proportion of Type 15 variation is just a bit higher than that reported in other studies. Lee et al. argues that Type 15 variation is a contraindication for donor hepatectomy [22]. In the original classification, all forms including branching of the right anterior portal vein from the left portal vein or from anywhere on the umbilical port were discussed under Type 15 anatomy. In our series, we did not find the right anterior portal vein arisen from the umbilical port. In conclusion, none of the potential donors was excluded because of variations of portal vein in the donor elimination process. Hence, we think that our results can be transcribed into Turkish population.

### 4.3. Anomalies of Hepatic Artery

Insufficient venous drainage from the graft leads to congestion and graft damage resulting from increased portal blood flow, and the survey of the graft is remarkably shortened. Therefore, preoperative recognition of the anatomy of hepatic venous system and knowledge about likely variations remarkably increases the rate of success via changes in surgical strategies [23]. The number of the published articles about hepatic venous anatomy is few, and there is not any universal protocol to define its anatomy. All modern imaging methods can be used. Only a few studies were published in the medical literature by now. Most of the studies based on the findings seen on preoperative imaging methods. In our study, intraoperative ultrasonography images as well as preoperative imaging methods were used. Since our study was conducted with a specific population, that is, liver donors, it seems to have limitations in comparison with other published studies including subjects with a wide spectrum of diseases. However, we think that our series has yielded the most accurate results, since liver malignancies or benign lesions of the liver cause alterations in the venous anatomy of the liver due to several factors, such as infiltration and pressure. Hepatic venous anatomy is best displayed with 3D-CT in the axial plane [24]. We used modified classification described by Soyer et al. to categorize it. As a consequence of our study, the right hepatic vein was viewed in 496 of 496 liver donors. The right hepatic vein was formed as a single main trunk in 194 subjects (39.1%). Two right hepatic veins joined together and formed the right hepatic vein in 235 subjects (47.5%). In 53 donors (10.7%), three hepatic veins united at the same level as a single orifice and drained into the vena cava inferior (VCI). The middle hepatic vein was also viewed in all donors. A single main middle hepatic vein drained into the VCI in 163 (32.9%) of 496 donors; two hepatic veins drained into VCI joining together to form a single middle hepatic vein in 202 (40.7%) donors. In 70 donors (14.1%), three middle hepatic veins drained into VCI, joining as a single orifice; two separate middle hepatic veins drained into VCI in 61 (12.3%) donors. The left hepatic vein was also viewed in all donors, like other hepatic veins. The left hepatic vein existed as a single trunk distally giving rise to small branches in 129 donors (26%); two distinct left hepatic veins drained into VCI in 24 donors (4.8%). Two hepatic veins joined together and formed a single main trunk in 193 (38.9%) of 496 donors; three left hepatic veins drained into VCI at the same level as a single orifice in 150 donors (30.2%). In 315 donors (63.5%), the middle and left hepatic veins drained into VCI, uniting as a single orifice; the left and middle hepatic veins drained into VCI individually in 181 donors (36.5%). Soyer et al. [24] made the classification in 69 patients. Nelson et al. [25] reported results similar to the rates defined by Soyer. In the Soyer's classification, drainage of the middle hepatic vein into VCI as two separate hepatic veins was not described. However, in our series, 61 donors had this type of variant. Normal anatomy was described as the drainage of the hepatic veins into VCI as three main trunks appearing the letter “W.” Soyer et al. reported the ratio of normal anatomy to be 68%; Lafortune et al. [26] reported the same ratio to be 70%. In our series, normal anatomy (drainage into VCI like W) was observed in 356 (71.7%) of 496 subjects, this ratio is consistent with the ratios reported in the literature.

### 4.4. Anomalies of Hepatic Artery

The debate about the test that should be chosen to display the anatomy of bile tree in the preoperative period is still being continued [27]. A group of authors, including us, uses intraoperative cholangiography to image central bile ducts. Although intraoperative cholangiography possesses certain risks (allergic reactions, although the risk is low, radioactive exposure, and most importantly, cease of operation because of an unexpected biliary anatomy), in our series, we did not face to any case requiring any strategic change in the surgical technique or to stop the operation.

In our series, we used Couinaud classification that is the most widely used one for anatomic classification of bile tree [12, 28]. As described in the literature, 60–65 percent of normal population have normal biliary anatomy, in which the right and the left hepatic ducts form the main hepatic duct, uniting as two main canals [29]. As a result of our series, typical anatomy (type A) was observed in a lower rate (49.4%) in comparison with the literature data. We found trifurcation anatomy (type B), in which the right anterior and the right posterior hepatic ducts join together with the left hepatic duct at the same level, in a rate (12%) equal to that in the original classification. In our series, type D biliary variation (ectopic union of the right sectoral ducts with the left hepatic duct) was more common (11.6%), in contrast to Couinaud's classification. Type C biliary anatomy (ectopic union of the right sectoral duct with the main hepatic duct) was found in a lower rate (15.7%). In the original classification, the rate of type C variation was reported to be 20%; the ratio of type D anatomy was reported to be 6%. In our series, type E variation, in which the right and the left main hepatic ducts are not formed, was found in a rate (4%) almost equal to that in the original classification. 

The type of variation that should be particularly emphasized is the type F. Its rate was 2% in Couinaud's classification, whilst in our series, it was observed in a higher rate (6%). Type F is described as the union of the right posterior bile duct with the cystic duct. It is the type of variation that must be paid attention especially by the surgeons dealing with laparoscopic biliary surgery. Otherwise, it may easily be stringed by mistake instead of cystic duct. Therefore, it is of particular importance among the reasons of iatrogenic damage to bile ducts. Biliary variations were found in 30 (27.7%) of 108 patients in the study conducted by Lee et al. and in 72 (34%) of 210 patients in the study conducted by Cheng et al. [29, 30]. In our series, we found biliary variations in a higher rate, in 201 (50.6%) of 398 donors. In the light of these results, it can be commented without a doubt that the incidence of biliary variations is higher in Turkish population.

### 4.5. Anomalies of Hepatic Artery

When the statistical analyses of the data were done, a considerable correlation was found between the distributions of bile ducts and portal vein (*P* : 0.019). The association between portal venous system and biliary system variants can be explained by embryologic development. During embryologic growth, the intrahepatic biliary ducts, that is, ductal plates, originated from the liver progenitor cells and the portal mesenchymal tissue are interweaved. Remodeling of ductal plate occurs within periportal mesenchymal tissue, which subsequently forms the portal vein. After that phase, the growths of bile ducts and portal vein become associated with each other. That the variations of portal vein are common particularly in the patients with biliary atresia is another finding supporting that correlation [31, 32].

##  Conflict of Interests

The authors do not have any financial relationship with the organization that sponsored the research.

## Figures and Tables

**Table 1:** The results in variations of bile ducts.

Type	Description	Our results	
A	The normal biliary anatomy	197 (49,4%)	
B	Trifurcation (The right anterior and the right posterior hepatic ducts join together with the left hepatic duct at the same level)	49 (12,3%)	
C	The ectopic union of right sectoral duct with the main hepatic duct		
C1		40 (10%)	
C2		23 (5,7%)	
D	The ectopic union of right sectoral ducts with the left hepatic duct		
D1		36 (9,4%)	
D2		9 (2,2%)	
E	The right and the left main hepatic ducts are not formed	20 (5%)	
F	The union of the right posterior bile duct with the cystic duct	24 (6%)	

Total	398	100%	

Biliary system was categorized according to the Couinaud classification [12].

**Table 2:** Hepatic artery series; comparison between the results of our series and others.

Type	Hiatt et al. [16] (*N*: 1000)	Gruttadauria et al. [14] (*N*: 701)	Our results (*N*: 496)	Rygaard [16] (*N*: 216)	Michels [13] (*N*: 200)	Daly [16] (*N*: 200)
1	75,7%	57,7%	**64,5%**	75,5%	55%	76%
2	9,7%	11,55%	**9,5%**	4,6%	18%	7,7%
3	10,6%	14,98%	**13,1%**	13,4%	18%	12%
4	2,3%	7,42%	**4%**	1,9%	4%	0%
5	1,5%	0,86%	**1%**	1,4%	2,5%	0%

The others	0,2%	7,62%	**7,9%**	3,2%	0,5%	6%

Arterial system was categorized according to the modified Michels' classification [16].
